# Fatal SARS-CoV-2 Infection among Children, Japan, January–September 2022^1^

**DOI:** 10.3201/eid3008.240031

**Published:** 2024-08

**Authors:** Shingo Mitsushima, Yuichiro Yahata, Yuuki Tsuchihashi, Chiaki Ikenoue, Munehisa Fukusumi, Shogo Otake, Tomoe Shimada, Takuya Yamagishi, Hajime Kamiya, Yusuke Kobayashi, Hitomi Kurosu, Mai Okuyama, Saeko Morino, Miho Shibamura, Sayaka Takanashi, Taro Kamigaki, Kanako Otani, Manami Nakashita, Hanae Ito, Ayako Miyazaki, Masaya Koshiko, Masanao Harakuni, Megumi Onuma, Hiroko Chiba, Maki Masutani, Natsu Sudani, Yuka Satsuki, Taketoshi Takara, Akira Oka, Tomohiro Katsuta, Naoki Shimizu, Akihiko Saitoh, Tetsuya Sakamoto, Motoi Suzuki, Tomimasa Sunagawa, COVID-19 Fatal Pediatric Cases Database Group

**Affiliations:** National Institute of Infectious Diseases, Tokyo, Japan (S. Mitsushima, Y. Yahata, Y. Tsuchihashi, C. Ikenoue, M. Fukusumi, S. Otake, T. Shimada, T. Yamagishi, H. Kamiya, Y. Kobayashi, H. Kurosu, M. Okuyama, S. Morino, M. Shibamura, S. Takanashi, T. Kamigaki, K. Otani, M. Nakashita, H. Ito, A. Miyazaki, M. Koshiko, M. Harakuni, M. Onuma, H. Chiba, M. Masutani, N. Sudani, Y. Satsuki, T. Takara, M. Suzuki, T. Sunagawa);; Saitama Children’s Medical Center, Saitama, Japan (A. Oka); St. Marianna University School of Medicine, Kanagawa, Japan (T. Katsuta, N. Shimizu);; Niigata University Graduate School of Medical and Dental Sciences, Niigata, Japan (A. Saitoh);; Teikyo University School of Medicine, Tokyo (T. Sakamoto)

**Keywords:** COVID-19, 2019 novel coronavirus disease, coronavirus disease, severe acute respiratory syndrome coronavirus 2, SARS-CoV-2, viruses, respiratory infections, zoonoses, pediatrics, children, death, encephalopathy, myocarditis, underlying disease, Japan

## Abstract

To determine the characteristics of pediatric patients 0–19 years of age who died after onset of SARS-CoV-2 infection in Japan during January 1–September 30, 2022, we reviewed multiple sources. We identified 62 cases, collected detailed information from medical records and death certificates, and conducted interviews, resulting in 53 patients with detailed information for our study. Among 46 patients with internal causes of death (i.e., not external causes such as trauma), 15% were <1 year of age, 59% had no underlying disease, and 88% eligible for vaccination were unvaccinated. Nonrespiratory symptoms were more common than respiratory symptoms. Out-of-hospital cardiac arrest affected 46% of patients, and time from symptom onset to death was <7 days for 77%. Main suspected causes of death were central nervous system abnormalities (35%) and cardiac abnormalities (20%). We recommend careful follow-up of pediatric patients after SARS-CoV-2 infection during the first week after symptom onset, regardless of underlying diseases.

COVID-19 has spread worldwide since December 2019. Among the mutated variants, some were designated by the World Health Organization as variants of concern because of their transmissibility or virulence (*1*). On November 26, 2021, the Omicron variant was designated as one such variant of concern (*2*). The first case caused by the Omicron variant was detected in November 2021 in South Africa, and the variant quickly spread worldwide, including to Japan (*3*). In Japan, the proportion of the Omicron variant among detected in SARS-CoV-2 isolates rose sharply in late December 2021, reached almost 100% in January 2022, and remained almost 100% throughout our study period (*4*).

In Japan, public health centers and hospitals mandatorily registered the number of COVID-19 cases and the characteristics of those cases at diagnosis (e.g., date of symptom onset and severity) on the Health Center Real-time Information-sharing System on COVID-19 (HER-SYS), a national surveillance system. Public health centers next conducted epidemiologic investigations based on information in HER-SYS. HER-SYS did not require mandatory follow-up of the details of the clinical course (e.g., medical intervention, hospitalization, and death) and therefore could not collect accurate information about severe or fatal cases.

After the Omicron variant was first detected, in 2022, the number of pediatric COVID-19 patients increased dramatically. According to national data, by December 2021 the number of SARS-CoV-2–positive patients 0–19 years of age was 240,000 (0–9 years, 84,000; 10–19 years, 156,000); during January–September 2022 (the Omicron variant period), the number increased to 4.8 million (0–9 years, 2.4 million; 10–19 years, 2.4 million) (*5*). The trend of increasing pediatric patients, including those hospitalized as Omicron began to spread, was also observed in South Africa (*6*,*7*). Although the number of reported deaths among patients 0–19 years of age was only 3 in 2020–2021, starting in January 2022 the number increased as the number SARS-CoV-2–positive patients 0–19 years of age increased (*5*). 

In general, SARS-CoV-2 infections in children are considered to be less severe than those in adults, and deaths among children are rare (*8*). Few studies have reported the details of fatal cases in children after SARS-CoV-2 infection, such as clinical courses leading to death and causes of death, especially during the Omicron variant period. Therefore, we aimed to determine the characteristics of fatal cases in children after SARS-CoV-2 infection during that period. However, we could not determine the exact number or the details, including cause of death (COD), based on the national surveillance system. Thus, we identified fatal cases in children through multiple sources and describe the overall characteristics and clinical courses, categorized by suspected COD, for pediatric patients after SARS-CoV-2 infection during the Omicron variant period. Our field investigation was conducted under the stipulations of the Infectious Diseases Control Law; therefore, ethics approval was not needed for our study.

## Materials and Methods

In Japan, the Ministry of Health, Labour and Welfare (MHLW) and the National Institute of Infectious Diseases conducted an enhanced epidemiologic investigation in cooperation with academic societies (Japan Pediatric Society, The Japanese Society of Intensive Care Medicine, and the Japanese Association for Acute Medicine). They investigated fatal cases after SARS-CoV-2 infection in patients 0–19 years of age (fatal pediatric cases) under the Act on the Prevention of Infectious Diseases and Medical Care for Patients with Infectious Diseases (Infectious Diseases Control Law).

To determine the actual number and characteristics of the cases, we collected information on fatal pediatric cases from the MHLW, local governments, public health centers, HER-SYS, academic societies, and the media. A case was defined as a fatal case occurring after SARS-CoV-2 infection in a young patient 0–19 years of age whose date of symptom onset or death was during January 1–September 30, 2022. The diagnosis of SARS-CoV-2 infection was based on PCR or antigen test results. Because our study was based on a public health response requiring rapid results, the study period was set until the end of September 2022. We collected cases without considering the duration from day of symptom onset to day of death because we assessed patients, including those with a long-time course, from symptom onset to death.

During August–December 2022, the research staff of the National Institute of Infectious Diseases collected epidemiologic data from local health authorities, and 2 or 3 staff members, including at least 1 pediatrician, visited the hospitals where the patients died (field investigation). We also obtained descriptions of epidemiologic investigations conducted by the public health centers. We collected data on the characteristics of the patients from medical records and death certificates of the hospital where the patients died and from records of epidemiologic investigations conducted by the public health centers. We collected information on patient age, sex, body weight, height, gestational age, physical handicap, SARS-CoV-2 vaccination history of parents and patients, underlying diseases, clinical courses comprising data on the diagnostic examination, date of symptom onset, date of admission, date of first consultation, date of cardiopulmonary arrest, date of death, symptoms/findings, out-of-hospital cardiac arrest, suspected infection source, pathogen detected in blood culture, imaging findings, treatment, and suspected COD. We did not perform additional testing to determine the suspected COD, which we classified as internal or external; we defined internal COD as death caused by disease excluding trauma and external COD as death caused by trauma. Our classification was intended to distinguish between internal and external COD for which the association with SARS-CoV-2 infection was considered low (e.g., death caused by incidental trauma after SARS-CoV-2 infection). We also divided the internal COD into central nervous system (CNS) abnormalities, cardiac abnormalities, respiratory abnormalities, other, and unknown. We determined those categorizations by comprehensively reviewing medical records, death certificates, and our physician interviews. Next, we conducted descriptive epidemiology of the characteristics of the patients and calculated the median and interquartile range (IQR) for the number of days from symptom onset to cardiopulmonary arrest and for the number of days from symptom onset to death. To test for goodness of fit in selected variables, we performed χ^2^ or Fisher exact tests. To compare intervals between symptom onset and first consultation, cardiopulmonary arrest, or death among different initial CODs, we next performed the log-rank test. For all statistical tests we used R 4.3.1 (The R Foundation for Statistical Computing, https://www.r-project.org). For some cases, we could not describe the details of patients because of privacy reasons.

## Results

**Figure 1:**
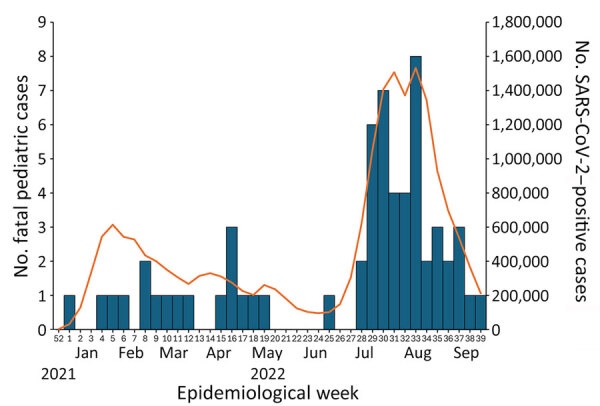
Number of reported fatal cases after COVID-19 among 61 children 0–19 years of age, by week of symptom onset or diagnosis, Japan, January–September 2022, and number of reported COVID-19 cases among patients of all ages. Excludes 1 patient for whom date of symptom onset and date of diagnosis of COVID-19 were unknown. Date of symptom onset for the 61 pediatric patients was January 1, 2022 (epidemiologic week 52, 2021) through September 30, 2022 (epidemiologic week 39, 2022). Bars denote the number of reported fatal pediatric cases (left axis); line denotes the numbers of COVID-19 patients of all ages reported by the Ministry of Health, Labour and Welfare (right axis). Scales for the y-axes differ substantially to underscore patterns but do not permit direct comparisons.

We identified 62 cases. Symptom onset or diagnosis of the first fatal pediatric case occurred in epidemiologic week 1 of 2022 (January 3–9), and subsequent cases occurred continuously (Figure 1). The number of cases increased on week 28 (July 11–17) and peaked during week 33 (August 15–21). The number of fatal cases seemed to be in line with the reported number of COVID-19 patients of all ages (*9*).

**Figure 2:**
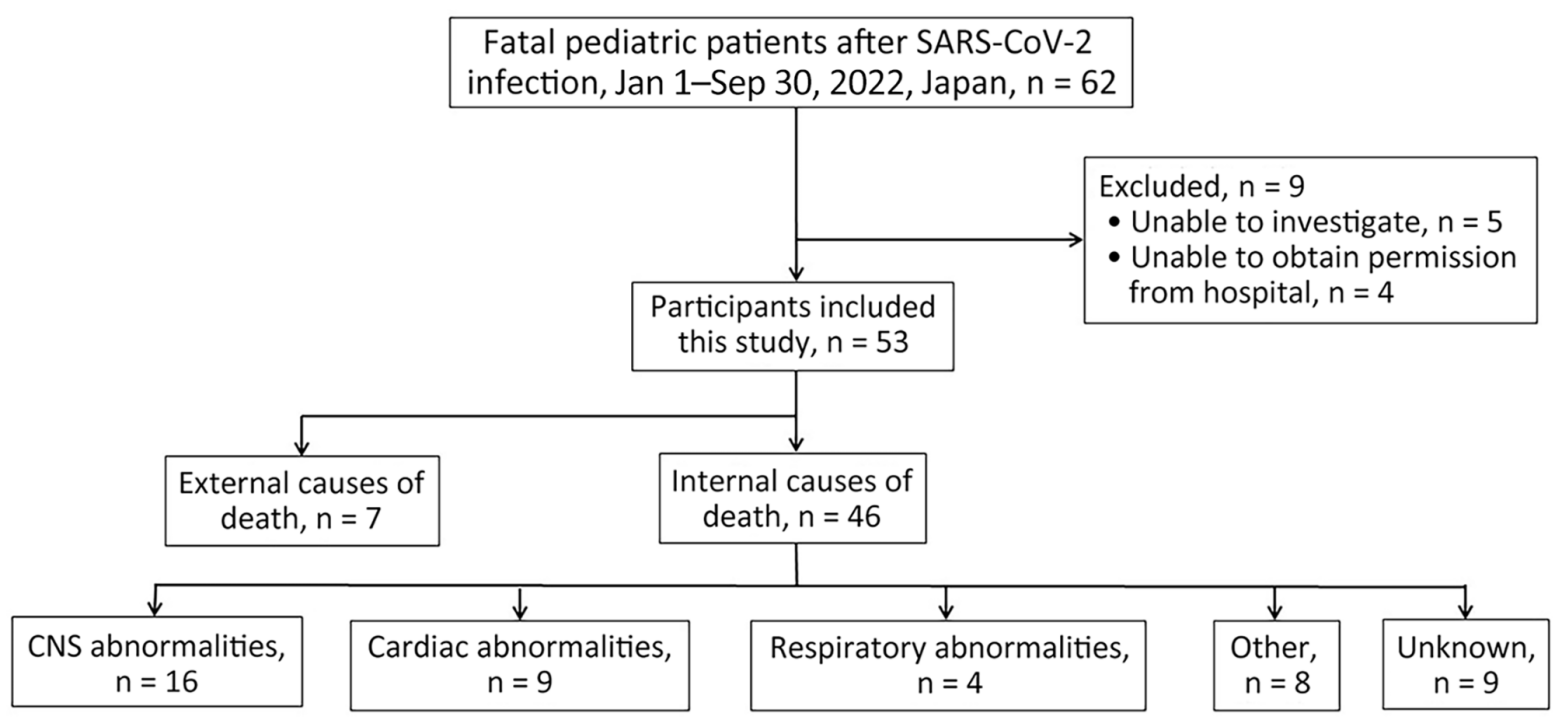
Patient selection in study of fatal SARS-CoV-2 infection among children, Japan, January–September 2022. CNS**,** central nervous system.

Of the 62 cases, we could not conduct field investigations for 5 (8%) and were unable to obtain permission to publish data from the hospital for 4 (6%). We thus describe the remaining 53 cases. Among suspected CODs, 46 (87%) were internal and 7 (13%) were external. The 46 internal COD cases were further divided (Figure 2).

### Fatal Pediatric Cases with Internal CODs 

Among the 46 patients with internal COD, most were 5–11 years of age, but deaths per 100,000 persons were highest among those <1 year of age (Appendix Table). Seven patients were <1 year of age (3 were <6 months of age), which was the highest number by age. No underlying diseases were noted for 27 patients, whereas some underlying diseases were noted for 19 patients. At the time of investigation, 24 (52%) patients were eligible for vaccination against SARS-CoV-2 because they were >5 years of age. Among them, 21 (88%) had received no vaccination, but the other 3 (13%) patients who were >12 years of age had received first and second vaccine doses, and their dates of symptom onset were >3 months after the date of their last vaccination. The suspected source of infection for 21 (46%) patients was within the family.

**Figure 3:**
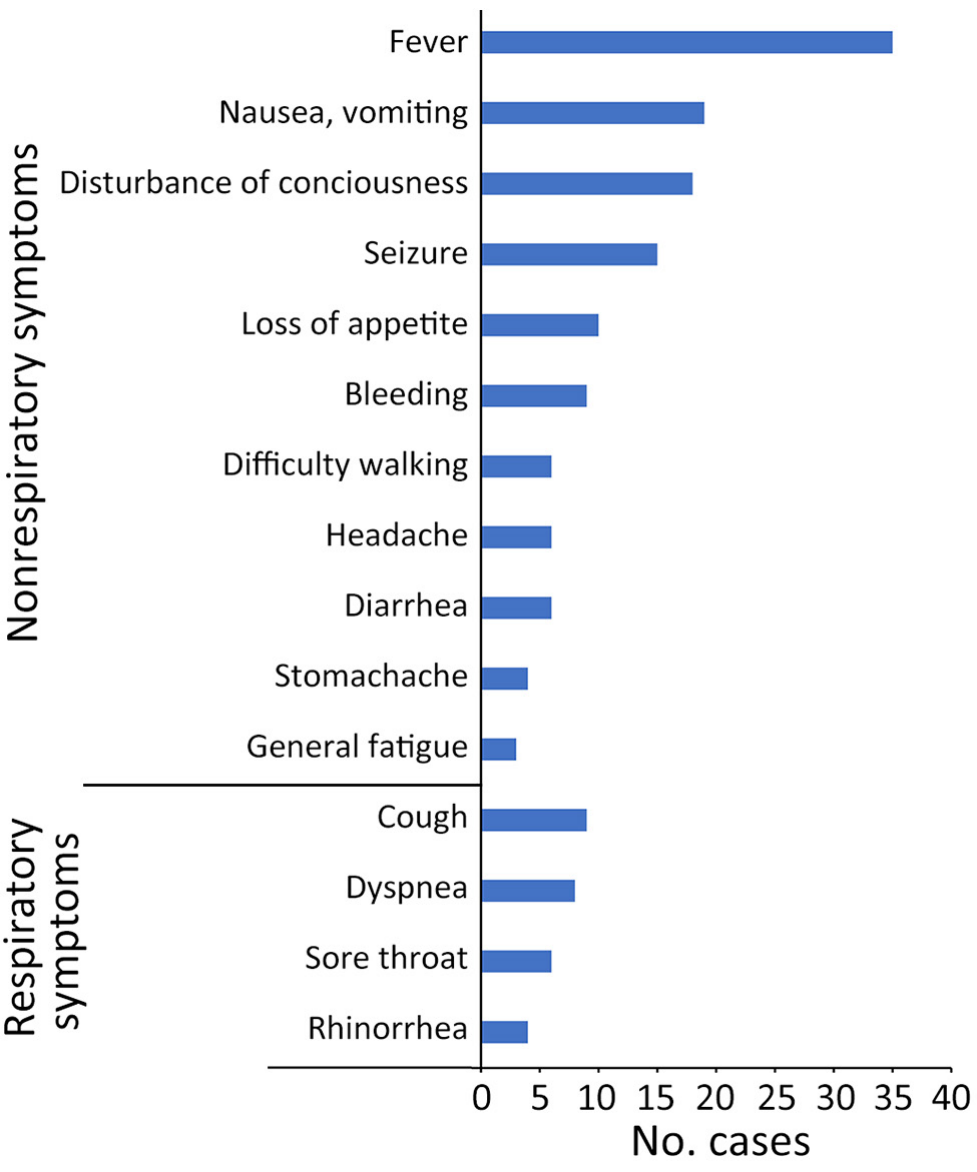
Distribution of symptoms before admission or death in outpatient settings in study of fatal SARS-CoV-2 infection among children, Japan, January–September 2022 (n = 46). Data account for multiple symptoms in some patients.

Before hospital admission, nonrespiratory symptoms were more common than respiratory symptoms (Figure 3). The most frequent suspected CODs were CNS abnormalities (e.g., acute encephalopathy), followed by cardiac abnormalities (e.g., acute myocarditis) and respiratory abnormalities (e.g., acute pneumonia). We did not find any instances of multisystem inflammatory syndrome in children. Among patients without underlying disease, the most common suspected COD was CNS abnormalities for 11, followed by cardiac abnormalities for 5; there were no cases of respiratory abnormalities. The number of deaths confirmed in the emergency department before the patient could be admitted to hospital was 19, and the number after admission was 27. Forty-six percent of patients died of out-of-hospital cardiac arrest. The proportion of out-of-hospital cardiac arrest caused by CNS abnormalities was significantly lower than that not caused by CNS abnormalities (p = 0.02), whereas the proportion of out-of-hospital cardiac arrest caused by cardiac abnormalities did not differ significantly from that not caused by cardiac abnormalities (p = 0.26).

We collected data on blood examinations, drugs, and treatment for 27 hospitalized patients (Table 1; Table 2). We excluded patients with external COD, those who died before they could be admitted, and those with out-of-hospital cardiac arrest (because the results of blood examinations in patients with cardiopulmonary arrest were extreme and reflected cardiac arrest and not a disease condition). D-dimer levels were high. Among patients for whom blood cultures were positive, we determined that for 1 patient, the pathogen detected from blood culture potentially contributed to death, whereas for the remaining patients, it was difficult to evaluate whether the pathogen affected death. Autopsies were performed for 7 patients, of which 2 showed no abnormalities, 2 showed evidence of pathology, and 3 showed inconclusive evidence.

**Table 1:** Results of blood examinations, drugs, and treatments after hospitalization for 27 patients in study of fatal SARS-CoV-2 infection among patients 0–19 years of age, Japan, January–September 2022*

Blood examinations at admission†	No. patients	Median (IQR)	Reference range
Leukocytes, no./μL	12	9,200 (6,270–14,450)	4,100–16,300
Neutrophils, %	11	65.7 (32.6–76.0)	18–94
Lymphocytes, %	11	27.0 (11.4–32.3)	18–82
Hemoglobin, g/dL	12	13.4 (12.8–14.5)	11.5–14.4
Hematocrit, %	12	41.2 (38.9–44.6)	34.5–43.0
Platelets, 10^4^/μL	12	19.4 (15.0–25.7)	18.0–51.0
PT-INR	5	1.32 (1.23–1.60)	0.75–1.15
Fibrinogen, mg/dL	6	186 (144–244)	200–400
D-dimer, ng/dL	5	39.3 (27.3–79.7)	0.15–1.0
Albumin, g/dL	10	4.6 (4.1–4.8)	3.6–4.7
Total bilirubin, mg/dL	7	0.31 (0.30–0.80)	0.3–0.9
Alanine aminotransferase, U/L	12	47 (25.5–274.5)	9–28
Aspartate aminotransferase, U/L	12	61 (42–322)	24–38
Lactate dehydrogenase, U/L	11	397 (254–738)	175–320
Creatine kinase, U/L	10	83.5 (62–201)	46–230
C-reactive protein, mg/dL	12	0.31 (0.11–0.47)	0.0–0.3
Sodium, mmol/L	12	137 (135–142)	137–144
Potassium, mmol	12	4.5 (3.7–5.3)	3.6–4.7
Glucose, mg/dL	9	171 (138–236)	70–200
Blood urea nitrogen, mg/dL	12	15.9 (13.8–19.5)	6.6–19.6
Creatinine, mg/dL	12	0.61 (0.42–1.38)	0.25–0.34
Lactate, mmol/L	9	4.8 (4.2–9.7)	0.5–2.0

*Reference ranges are for persons 6 years of age. IQR: interquartile range, PT-INR, prothrombin time international normalized ratio. †Excludes patients who died before admission to hospital and those with out-of-hospital cardiac arrest.

**Table 2:** Results of examinations, drugs, and treatments after hospitalization for 27 patients in study of fatal SARS-CoV-2 infection among patients 0–19 years of age, Japan, January–September 2022*


Result	No. (%) patients
Pathogen detected in blood culture†	
*Streptococcus* spp.	2 (7)
*Escherichia* spp.	1 (4)
*Fusobacterium* spp.	1 (4)
Chest CT findings‡	
Ground-glass opacity	5 (21)
Consolidation	5 (21)
Pleural effusion	1 (4)
Brain CT findings§	
Cerebral edema	14 (40)
Loss of gray-white matter differentiation	7 (20)
Intracranial hemorrhage	3 (9)
Hydrocephalus	3 (9)
Congenital abnormalities	3 (9)
Drug†	
Inotrope	16 (59)
Steroid	14 (52)
Antiviral	12 (44)
Treatment†	
Mechanical ventilation	23 (85)
Extracorporeal membrane oxygenation	5 (19)
Targeted temperature management	4 (15)
Blood transfusion	13 (48)
Immune globulin	4 (15)
Dialysis	3 (11)
Plasmapheresis	1 (4)

*CT, computed tomography. †Includes multiple pathogens, drugs, and treatments. ‡Includes multiple findings; 24 cases involving chest CT are included. §Includes multiple findings; 35 cases involving head CT are included.

#### CNS Abnormalities 

Of the 16 cases, CNS abnormalities were observed most often among patients 5–11 years of age, whereas none were observed in patients <1 year of age (Table 3). The most frequent clinical sign/symptom was fever, followed by disturbance of consciousness, seizure, nausea or vomiting, and headache, which are typically associated with acute encephalopathy. Digestive symptoms (e.g., stomachache and diarrhea) developed in a few patients. Acute encephalopathy affected 12 (75%) patients, intracranial hemorrhage in 1 (6%), and other conditions in 3 (19%). Among the 12 patients with acute encephalopathy, hemorrhagic shock and encephalopathy syndrome (HSES) was suspected for 5 (42%), encephalopathy with acute fulminant cerebral edema was present in 1 (8%), and unclassifiable encephalopathy was present in 6 (50%).

**Table 3:** Characteristics of 16 patients for whom central nervous system abnormalities were suspected of causing death in study of fatal SARS-CoV-2 infection among patients 0–19 years of age, Japan, January–September 2022*

Characteristics	Value
Age group, y	
0	0
1–4	5 (31)
5–11	9 (56)
12–19	2 (13)
Sex	
M	8 (50)
F	8 (50)
Underlying disease	
Yes	5 (31)
No	11 (69)
Vaccination	
None	11 (69)
Ineligible	5 (31)
Symptoms/signs	
Fever	14 (88)
Disturbance of consciousness	13 (81)
Seizure	11 (69)
Nausea/vomiting	8 (50)
Headache	6 (38)
OHCA	
Yes	3 (19)
No	13 (81)
Days from symptom onset to first consultation	
0–2	16 (100)
>3	0
Median (IQR)	0.5 (0–1.0)
Days from symptom onset to cardiopulmonary arrest	
0–4	11 (69)
5–9	0
>10	5 (31)
Median (IQR)	2.0 (1.0–12.0)
Days from symptom onset to death	
0–4	9 (56)
5–9	0
>10	7 (44)
Median (IQR)	3.5 (2.0–14.5)

*Values are no. (%) patients except as indicated. OHCA, out-of-hospital cardiac arrest, IQR, interquartile range.

Among 11 patients for whom time from symptom onset to cardiopulmonary arrest was <5 days, 6 (55%) had cerebral edema and 3 (27%) had rapidly progressing cerebral herniation. Of the 5 patients for whom the interval was >10 days, 4 (80%) showed a flat electroencephalogram in the first 4 days after symptom onset. Of the 16 patients who died of CNS abnormalities, 2 died before and 14 died after hospitalization.

#### Cardiac Abnormalities

The most frequent symptom was fever, followed by nausea or vomiting, loss of appetite, consciousness disturbance, and cough (Table 4). Out-of-hospital cardiac arrest affected 67% of the 9 patients with cardiac abnormality, higher than the 19% of patients for which the suspected COD was CNS abnormalities, although no significant difference was noted in the number of days from symptom onset to first consultation between the patients who died of cardiac abnormalities versus CNS abnormalities (p = 0.1). The number of days from symptom onset to cardiopulmonary arrest and death for all patients was <7 days, whereas no difference in intervals was noted between CNS abnormalities (p = 0.6) and cardiac abnormalities (p = 0.1). Clinical acute myocarditis was detected in 8 of the 9 patients.

**Table 4:** Characteristics of 9 patients for whom cardiac abnormalities were suspected as cause of death in study of fatal SARS-CoV-2 infection among patients 0–19 years of age, Japan, January–September 2022*

Characteristic	Value	
Sex		
M	2 (44)	
F	7 (56)	
Underlying disease		
Yes	4 (44)	
No	5 (56)	
Vaccination		
None	4 (44)	
2 doses	1 (11)	
Ineligible	4 (44)	
Symptoms/signs		
Fever	7 (78)	
Nausea/vomiting	4 (44)	
Loss of appetite	3 (33)	
Disturbance of consciousness	2 (22)	
Cough	2 (22)	
OHCA		
Yes	6 (67)	
No	3 (33)	
Days from symptom onset to first consultation	
0–2	7 (78)	
3–6	2 (22)	
>7	0	
Median (IQR)	1.0 (0–2.0)	
Days from symptom onset to cardiopulmonary arrest		
0–2	4 (44)	
3–6	5 (56)	
>7	0	
Median (IQR)	4.0 (2.0–4.0)	
Days from symptom onset to death		
0–2	4 (44)	
3–6	5 (56)	
>7	0	
Median (IQR)	4.0 (2.0–5.0)	

*Values are no. (%) except as indicated. Age distribution could not be shown because it could lead to the identification of specific persons. OHCA, out-of-hospital cardiac arrest; IQR, interquartile range.

### Fatal Pediatric Cases with External Causes of Death 

Of the 7 patients with an external COD, 2 were <5 years of age and 5 were >5 years of age. External CODs included unexpected accidents such as drowning and suffocation but not traffic accidents, fires, poisoning, or disasters. Some patients were suspected of experiencing consciousness disturbance before the external COD.

## Discussion

In our descriptive study, to present the overall characteristics and clinical course after SARS-CoV-2 infection, we investigated not only internal CODs but external CODs. Our study included 62 fatal pediatric cases for which dates of symptom onset and death were January 1–September 2022. The most common patient age was <1 year, similar to the trend reported for a prior study in the United States (*10*). More than half of the patients whose COD was internal had no underlying disease, and most were unvaccinated. CNS abnormalities comprised the highest number of suspected CODs, followed by cardiac abnormalities. We observed that nearly 80% of the patients died and 85% had cardiopulmonary arrest within 7 days of first symptoms, regardless of the suspected COD. That finding was consistent with that of a previous survey of deaths among children and young persons in England after SARS-CoV-2 infection during March 2020–February 2021, which reported that the time interval between a positive SARS-CoV-2 test and death in 84% for those dying of COVID-19 was within 7 days (*11*). Several patients in our study were transferred to the hospital soon after rapid illness progression and received intensive care but did not recover. Follow-up of pediatric cases in an out-of-hospital setting is crucial for the first 7 days after symptom onset.

We found more patients without than with underlying disease. Earlier reports, before the advent of the Omicron variant, indicated that deaths caused by COVID-19 tend to occur in those with underlying disease (*11*,*12*). A study conducted in South Korea, which included the Omicron variant period, showed that more than half of the patients <18 years of age who died had no underlying disease, which was similar to our finding (*13*). In addition, the proportion of nonrespiratory symptoms was higher than that of respiratory symptoms (Figure 3). The COVID-19 registry survey conducted by the Japan Pediatric Society during the very early Omicron variant phase (January 1–February 20, 2022) reported that among 1,058 pediatric patients with COVID-19, respiratory symptoms such as cough and runny nose were common (*14*), which differed from our findings, possibly because of differences in illness severity or in the study period and population. Our study showed the value of considering nonrespiratory symptoms (e.g., neurologic and digestive) as critical signs during the Omicron variant period.

Regarding vaccination, among the 46 case-patients with internal COD, 52% of the patients in our study were eligible for vaccination; among them, 13% had received 2 doses of vaccine. All vaccinated patients were *>*12 years of age. In Japan, vaccination against SARS-CoV-2 for those >12 years of age started in June 2021, that for children 5–11 years of age started in February 2022, and that for those 6 months to 4 years of age started in October 2022. Thus, in our study, patients >5 years of age were eligible for vaccination before disease onset. The percentage of children 5–11 years of age who had received >1 vaccine was 22.5% as of September 30, 2022, which was higher than the 13% of children in our study who had undergone vaccinations (*15*). Prior studies showed that COVID-19 vaccines for children 5–11 years of age could prevent severe COVID-19, including that caused by the Omicron variant (*16*–*18*). Although the lower proportion of persons vaccinated in our study population compared with the general population might have affected their deaths, we could not evaluate the effect of vaccination because we collected data on fatal cases only. Furthermore, caution is necessary when applying the results of our study to the general population because of the low proportion of vaccinated patients.

Among the 16 patients with CNS abnormalities, we collected data for 12 with acute encephalopathy. Among them, 5 patients had HSES, and 1 had encephalopathy with acute fulminant cerebral edema, which might be remarkable. In general, the most common type of encephalopathy is acute encephalopathy with biphasic seizures and late reduced diffusion, followed by clinically mild encephalitis/encephalopathy with a reversible splenial lesion and HSES (*19*,*20*). HSES is a rare type of encephalopathy with a high mortality rate. It is associated with cytokine storms with disseminated intravascular coagulation and multiple organ failure at the time of initial examination (*21*,*22*). In a prior study, encephalopathy with acute fulminant cerebral edema was reported to be relatively common among COVID-19 patients, and a type of encephalopathy in which initial examinations (e.g., blood tests and computed tomography of the head) showed only minor abnormalities and then rapid progression was reported (*21*,*23*). Other reports, which might include this particular encephalopathy, suggested that encephalopathy results in severe sequelae or fatal outcomes (*23*–*26*). The Japanese Society of Child Neurology and the Research Committee on Acute Encephalopathy conducted an epidemiologic investigation that suggested that acute encephalopathy associated with COVID-19 had a poor clinical outcome and was more severe than acute encephalopathy associated with other viruses (*23*,*27*). In our study, frequency of out-of-hospital cardiac arrest was significantly lower among patients who died of CNS abnormalities than among those who died of causes other than CNS abnormalities, whereas the intervals between symptom onset and cardiopulmonary arrest or death did not differ between patients who died of cardiac abnormalities or CNS abnormalities. Illness of pediatric patients who died of CNS abnormalities was assessed as less severe at the time of arrival, but both conditions worsened rapidly. For some patients, electroencephalogram was flat, which meant irreversible neurologic damage, within a few days after symptom onset. The irreversible neurologic damage might be one of the reasons why patients with neurologic complications are at risk of dying. Therefore, clinicians should be aware that COVID-19 may cause acute encephalopathy that leads to irreversible neurologic damage and that acute encephalopathy can rapidly progress to death even if the patient receives intensive care.

Among the strengths of our study is that we detected fatal pediatric cases for which outcomes were not reported to HER-SYS because we could collect cases from multiple sources. In addition, we could describe the details of the cases by suspected COD, which were not collected by HER-SYS. The insights provided by our study differ greatly from those that would be obtained from a database study.

The first limitation of our study was that we might not have captured all characteristics during the Omicron variant period because it concluded while the epidemic was still ongoing. Second, we did not examine the causal relationship between SARS-CoV-2 infection and death. Through review of medical records, death certificates, and our physician interviews by pediatricians, we were able to determine whether SARS-CoV-2 infection was an underlying COD. We determined that SARS-CoV-2 infection contributed to most of the deaths. However, there were a few cases in which underlying disease potentially contributed more to death than did SARS-CoV-2 infection. It was difficult to accurately evaluate the extent to which SARS-CoV-2 infection affected death in cases for which autopsies or examinations such as imaging and blood examinations could not be performed. Therefore, determining whether SARS-CoV-2 infection was the underlying COD was challenging. Third, cases might have been underreported because case detection was based on voluntary reporting. If a physician determined that there was no association between SARS-CoV-2 infection and death, the case might not have been reported. Encephalopathy or carditis might be more likely to have been reported, whereas reporting of deaths caused by trauma or exacerbation of underlying diseases after SARS-CoV-2 infection might be less likely. However, the study conducted in Korea showed similar epidemiologic trends, such as in the number of deaths and the age of patients, which support the results of our study (*13*). Fourth, collection of all data was not always possible because of missing data. Last, diagnosis, examination, and treatment might differ because they were conducted at different hospitals.

In conclusion, the most common COD among fatal pediatric SARS-CoV-2 cases in this study was CNS abnormalities, followed by cardiac abnormalities. For pediatric patients, clinicians should pay attention not only to respiratory signs/symptoms but also to nonrespiratory signs/symptoms. In addition to addressing signs/symptoms, clinicians should carefully observe pediatric patients during at least the first 7 days from onset of COVID-19 regardless of underlying diseases because most pediatric patients who died rapidly progressed to death within that period.

Appendix Additional information for study of fatal SARS-CoV-2 infection among children, Japan, January–September 2022.
