# Supplementary material for: Fatal SARS-CoV-2 Infection among Children, Japan, January–September 2022
Source: Emerg Infect Dis. 2024 Aug;30(8):1589–98. doi: 10.3201/eid3008.240031 (PMC11286078; doi:10.3201/eid3008.240031)
Supplement: Appendix — Additional information for study of fatal SARS-CoV-2 infection among children, Japan, January–September 2022. [file 24-0031-Techapp-s1.pdf]

Article DOI: https://doi.org/10.3201/eid3008.240031

Fatal SARS-CoV-2 Infection Among Children, Japan, January–September 2022

Appendix

Appendix Table. Characteristics of fatal cases in 46 patients with internal causes of death in study of fatal SARS-CoV-2 Infection among patients 0–19 years of age, Japan, January–September 2022*

Characteristics	Value
Age group, y (n = 46)	
<1	7 (15)
1–4	15 (33)
5–11	18 (39)
12–19	6 (13)
Age group, y, no./100,000 persons	
<1	0.877
1–4	0.435
5–11	0.256
12–19	0.069
Sex (n = 46)	
M	23 (50)
F	23 (50)
Underlying disease (n = 46)	
Yes	19 (41)
No	27 (59)
Vaccination (n = 46)	
None	21 (46)
2 doses	3 (7)
Ineligible (<5 y of age)	22 (48)
Gestational age (n = 7)†	
Before term (30–36 wk)	4 (57)
Full-term (37–41 wk)	2 (29)
Unknown	1 (14)
Body weight (n = 46)	
Low‡	10 (22)
Normal	19 (41)
Excessive§	1 (2)
Unknown	16 (35)
Physical handicap (n = 46)	
Yes	11 (24)
No	33 (72)
Unknown	2 (4)
OHCA (n = 46)	
Yes	21 (46)
No	25 (54)
Diagnostic examination	
PCR	26 (57)
Antigen test	17 (37)
Unknown	3 (7)
Suspected causes of death (n = 46)	
Central nervous system abnormalities	16 (35)
Cardiac abnormalities	9 (20)
Respiratory abnormalities	4 (9)
MIS-C	0
Other	8 (17)
Unknown	9 (20)
Days from symptom onset to first consultation (n = 44)¶	
0–2	40 (91)
3–6	4 (9)
≥7	0
Median (IQR)	1.0 (0–1.0)
Days from symptom onset to cardiopulmonary arrest (n = 44)¶	
0–2	24 (55)
3–6	13 (30)
≥7	7 (16)

Characteristics	Value
Median (IQR)	2.0 (1.0–5.0)
Days from symptom onset to death (n = 44)¶	
0–2	21 (48)
3–6	13 (30)
≥7	10 (23)
Median (IQR)	3.0 (1.0–6.0)

*Values are no. (%) except as indicated. IQR, interquartile range; MIS-C, multisystem inflammatory syndrome in children; OHCA, out-of-hospital cardiac arrest.
†Seven patients <1 y of age were included.
‡Low body weight was defined as −2 SD from standard weight in patients <18 y of age or body mass index (BMI) <18.5 kg/m^2 in those aged 18 and 19 y.
§Excessive body weight was defined as +2 SD from standard weight in patients <18 y old or BMI >25.0 kg/m^2 in those aged 18 and 19 y.
¶Patients whose date of symptom onset was uncertain are excluded.
